# "The Hospital" Medico-Sociological Supplement (No. IV.)

**Published:** 1911-04-08

**Authors:** 


### "THE HOSPITAL" MEDICO-SOCIOLOGICAL SUPPLEMENT

(No. IV.)

### THE SOCIAL SIDE OF THE TREATMENT OF CONSUMPTION.

It is within the experience of those who have some general knowledge of the workings of present day philanthropy that, at any given period, there is a tendency for charity to be exercised in a marked degree in a particular direction: efforts are made in a way in which they were not made a few years ago and in which they may not be made a few years hence. The poor we have always with us, and there are many forms of assistance which do not fail; but there is a fashion in charity as in other things, and it is the business of those who have at heart the efficiency of charitable effort to see that the flood of human kindness, poured out so freely, should not only reach a high-water mark, but should flow in the most helpful channel. There can be little doubt that the prevention and treatment of consumption is one of the most prominent features of the charity of to-day. Schemes for the prevention of the disease, for the building of institutions for its treatment, for the education of the people in its significance, are constantly brought to our notice. The flood is with us: how is it to be directed?

### The Medical Aspect of the Subject.

This article is an attempt to draw attention to one side of the subject of the treatment of consumption. On the medical side, the problem is attacked daily: there is perhaps a tendency that the social side should be forgotten. Those who are brought into daily contact with poor consumptives, who visit them in their homes, who see how they live, who learn their point of view, cannot underestimate the social side of the disease. The medical terms in which the condition of the patient's lungs have been described may mean little to them, but they do know what is the patient's work and how he lives at home: they can form some judgment of his character and some opinion as to how far, apart from the medical aspect, he is likely to prove amenable to advice and treatment; they can estimate in some degree what is the ultimate chance of a cure. They find that consumption is no sudden disease actively infectious for a short period which may safely be forgotten when that period is over: on the contrary its attack is insidious, and it is hard to discover in its early stages, so that the unhappy victim, almost before he is aware of it, is found to be in too advanced a stage for effective treatment. Consumption clings to the four walls of a room: the Charity Organisation Society of New York City has described in striking terms how family after family in succession has been infected by living in certain dwellings in that city. Consumption is primarily the disease neither of childhood nor old age: it attacks the young working man and woman, the man and woman who can least be spared. The number of the social aspects of the disease can scarcely be limited.

If it is true that these social aspects exist, it must follow that in the treatment of consumption they must not be forgotten: to treat a working man for the disease without consideration of his home, his family, his work, and his mode of life, must prove ineffective. How far, then, do existing methods of treating the poor take into account the social side of consumption?

### Home Treatment.

The working man who is able to call in a doctor may be well or ill served: it cannot be expected that the sixpenny doctor so well known in the poorer parts of London should be in a position to give any particular attention to the case. His club doctor, if he has one, is a busy man dealing daily with a large number of trivial ailments: he will very likely advise treatment at a chest hospital. Over 27,000 new out-patients found their way to the chest hospitals of London in 1909, the great majority of the poor in whom the characteristic symptoms and physical signs of consumption had appeared. In the out-patient department skilled medical treatment will be obtained, but under conditions which make it difficult to take the social aspect of the case into consideration. There is no possibility of a visit at the home, there is scarcely an opportunity even for questioning and advising the patient himself, the crowds in the out-patient department are too great. If treatment in the hospital is advised, the physician is not allowed to select the most suitable case, but the patient is sent off to beg from subscribers for an in-patient letter. It may take him many weeks to obtain a letter: he may not obtain one at all; in any case he will have to wait a considerable time for admission.

### Hospitals and Their Scope.

Can it be maintained that the consumption hospitals, excellent as their work is, are doing all that is possible to cope effectually with the disease among the poor? I do not think that anyone can claim that there is no room for reform. At present the social side of the disease cannot adequately be taken into consideration: the hospitals themselves are working for the most part as independent units without any system of co-operation: continuous observation of the patient, on which the curative treatment so much depends, is scarcely possible, and it can hardly be said that hospital treatment for consumption in the comparatively unwholesome atmosphere of so large a city as London is an ideal arrangement.

### The Scottish Example.

Is there any better way? Edinburgh has discovered a way out. Many of the shortcomings referred to have been overcome in the anti-tuberculosis dispensary scheme inaugurated by Dr. Philip, in Edinburgh, in 1887. Paddington has now a dispensary on the same lines, Kensington and St. Marylebone have followed suit, one will shortly be opened in Stepney, and there are hopes of the establishment of similar institutions not only in other parts of London, but also in the provinces. The whole object and aim of these dispensaries is to take into full account the social side of the disease, to work not in competition but in co-operation with every existing hospital, charitable agency, sanatorium and other institution which will assist not only in the cure of the disease but in its prevention. A specialist in chest diseases is in charge of each dispensary, he has time carefully to examine each patient and to visit his home, and he is assisted by a nurse who by regular visiting can secure that the home conditions are as good as possible.

### Food and Shelter.

If extra nourishment is wanted, some charitable agency is asked to supply it; if hospital treatment is required, a letter is obtained and the dangerous waiting time is carefully bridged over; if sanatorium treatment is wanted, the Charity Organisation Society will supply it; if a shelter in the back garden is required, the shelter is found: moreover, the children are carefully examined from time to time, others who live with the patient are looked to, and so contact cases are discovered in such an early stage that they can be effectively dealt with. It may be objected that a system such as this must deprive the local practitioner of his patients, but one of the first rules of a dispensary is that each patient on his first attendance should be asked for his doctor's name: the doctor is at once communicated with, asking if his patient may attend the dispensary. Experience shows that local doctors do not object, but themselves send many patients to the dispensary, finding an expert opinion very valuable.

The dispensary, then, does not supersede existing institutions: it uses them all. It is true that it may to an extent intrude on a hospital out-patient department, but there is reason to hope that the out-patient department of the future will be run on more scientific lines than in the past: in other words, prevention as well as cure will find a place there, it will cease to compete, and begin to co-operate, its working methods will, in fact, approximate to those of the new dispensaries. How this may be done has already been indicated in a paper issued by the Committee which has recently been formed to raise funds for the provision of anti-tuberculosis dispensaries in the poorer parts of London.

### Sanatorium Treatment and the C.O.S.

Attention may be drawn to one other small but important feature in connection with the treatment of the consumptive poor of London. The Charity Organisation Society has for the past eight years sent suitable cases away for sanatorium treatment. The Jewish Board of Guardians does excellent work in the same direction, but the activities of the C.O.S. are not confined to any race or creed, and its forty-two district committees supply sanatorium cases from every part of London. The whole method of selection and treatment of patients is as scientific as it is possible to devise. The patient requires no recommendation beyond a good chance of recovery, a matter in which character and environment, as well as the condition of his lungs, have to be taken into account: he is passed by a medical referee, and goes with the minimum of delay to a sanatorium where he remains as long as the doctor wishes: his family is maintained, if necessary, while he is away, and, on his return, efforts are made to find him suitable work or to improve the conditions under which he must work. Three hundred and eighty-five persons had been sent away up to December 31, 1909, the average stay was seventeen weeks, and over 50 per cent. are now working or fit for work. At present, over 100 persons are sent away annually to sanatoriums, and the numbers steadily increase. The work is but a drop in the ocean, but is part of the Society's regular routine work of fitting the treatment to the case. It need hardly be added that the Society works in the closest co-operation with the new anti-tuberculosis dispensaries, as well as with hospitals and medical men generally. The social side of every case is carefully considered, and the aid of other agencies is invoked to rectify what is wrong: the medical officer of health, the guardians, the local health society or visitor, the clergy, all are asked to do their share.

### The Needs of the Moment.

What, then, is wanted, is a due recognition of the importance of the social side of the disease and of the necessity of co-operation in grappling with it. Ignorance still remains a powerful factor in the problem, but the energetic propaganda of the National Association for the Prevention of Consumption is having its effect. Other movements tend also to an improvement of the conditions under which the people live: better housing and a cleaner milk and meat supply are helpful factors in the fight. The recently published report of the Public Health Committee of the London County Council for 1909 shows a conspicuous fall in the death-rate from tuberculosis, but that is no reason why the fall should not be still more accelerated.

This article is written by a Londoner, and is mainly based on London experience, but the statements in it should apply, I think, to many places outside the Metropolis. The basis of the problem must remain the same: the remedy, if there be any value in the suggestions I have made, should be, in its main lines, the same also.

---

Nearly £3,000 has been raised for the new Edward Ward at the Royal Surrey County Hospital, and the cost of furnishing is being met by a draft on the capital fund of £2,200. The maintenance of the Edward Ward will cost £900 a year, and the additional £260 in annual subscriptions raised recently on its account, plus the capital since subscribed, is expected to maintain it for four years.

### PREVENTABLE SOCIAL ILLS.

### I.—THE PARENTHOOD OF THE UNWORTHY.

The examination of certain facts will suffice to show that our modern humane civilisation has unaware drifted into the position of a forcing-ground for the propagation of persons unfit to become parents, and for the protection and survival of their unfit offspring until such time as they also may be ready to take up their rôle of increasing the nation's bad breeding-stock. Dr. Archdall Reid's recent valuable researches into the question of heredity have given ground for the hope that the range of those unfit to become parents may be rather more limited than was at one time supposed. If the effects of under-feeding, of squalid surroundings, and even of physical defects are intransmissible to the offspring, there is reason to believe that from the foulest slums and most unpromising parentage a race of unimpaired vigour may be developed when the defects of the parental environment have been corrected. But so constant is the heritage of the epileptic, the insane, and the morally and intellectually defective, that improvement in the environment produces no corresponding response.

### Defective Children.

The defective child, however admirably trained, develops into an irresponsible man or woman, dangerous to self and society, only too ready for parentage, and practically certain to transmit the same inherited defects to descendants. The following pitiful tale of genealogy, extracted from the recently issued report of the Lancashire and Cheshire Society for the Permanent Care of the Feeble-minded, exhibits the rate of increase at which we are propagating the unfit:—

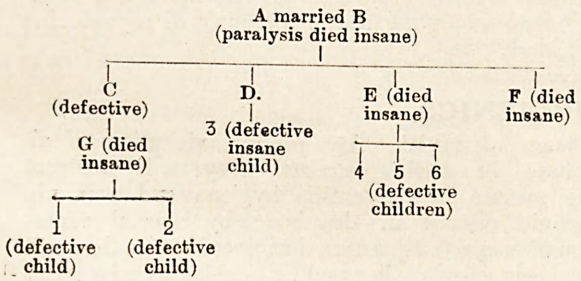

Thus from the marriage of two unfit persons we have eleven descendants, of whom one only (D) proved capable of maintaining himself, while six defective children are thrown on the world ready to carry on this disastrous sequence. The case of the six is more tragic than even that of their parents. For, owing to the awakened conscience of the nation, they are receiving a costly training in special schools adapted to develop as far as is possible their dulled faculties. Then from the safe shelter of school or institution they will pass to a vain attempt to compete for employment with normal men and women. The girls will fall a ready prey to temptation, and drift to a tramp life or the ignominy of the streets. For the boys there is the steady descent to the unemployable class, with variations in routine from workhouse to prison, ending probably in the asylum, but leaving a trail of illegitimate offspring, which the community adopts and prepares for following the father's career.

In a more primitive state of society, men, women and children who fall below the normal in intelligence perish from sheer inability to procure for themselves the necessaries of life. Feeble-minded children are often weakly and always require special care in the upbringing lest they should injure themselves. A very little neglect suffices to kill them, and countless defectives do perish thus, either soon after birth or during childhood, for the defective mother can never be taught the duties of motherhood, and it is among this class that babies are ignorantly fed on steak and onions or overlaid at night, or left to catch fire, or sacrificed in a hundred blundering ways. This short way with the defectives is not, however, recognised as tolerable in a civilised State. Even the unborn citizen, so soon as life stirs, has its rights, and none is so pitiful a failure that he may not claim food and shelter.

### The Increase in Defectives.

The increase of the defective type in modern communities is spreading till it threatens the stability of the entire nation. It has been computed that 60,000 children alone are in need of the special school accommodation, which the authorities are now empowered to furnish for their training. In very few localities have the necessary powers been used, and the reason is not far to seek. The very best successes of the special school are still unfit for the business of life. They learn slowly and forget all too soon. The only possible solution of the problem is segregation, under conditions which can shield them from becoming parents and utilise their feeble powers for their own good. It is a gigantic task, the segregation of 60,000 in one generation. So desperate a remedy does it appear that the process of "laisser-faire" is by common consent preferred, and before thirty years has passed the number will be doubled. Sooner or later, as they become actively troublesome, the greater proportion of these retrograded children will be provided for in workhouse, prison, or asylum. The public is accustomed to this expense, and the ever-swelling burden of the degenerates presented under this form is not resented. A comprehensive scheme, however, for the segregation of the feeble-minded would meet with a general outcry.

Is there no sign of hope? It is as usual through voluntary effort that the first symptoms of reform are appearing. The great work of ascertaining the extent of the evil has been thoroughly well performed, and in this pioneer work the name of Miss Dendy should be honoured. To her and to her colleagues in the Lancashire and Cheshire Society for the Permanent Care of the Feeble-minded we owe the development of that preventive treatment known as the colony system. Parallel with the admirable experiments at Sandlebridge, Dr. Fernald has been doing marvellous work for the feeble-minded at

Waverley, near Boston. These two institutions in England and the United States may be said to have removed the gravest difficulties in the way of segregation by demonstrating that the life can be made a happy and useful one. No legislation for the defectives could have succeeded, unless the ground had first been broken by voluntary enthusiasm experimenting on perfectly free lines. Another voluntary society has been formed in Yorkshire with similar aims. Hundreds of zealous workers are ready to follow suit, and everywhere there is evidence of a strong movement towards reform. Two difficulties are holding back the voluntary workers, and we venture to urge that both are readily removable without placing so great a strain on the nation as a measure for the compulsory segregation of the entire body of the feeble-minded.

The first difficulty, of course, is finance. At Sandlebridge the gross cost per head is £26. A Government grant is obtained for the day school work, and a few inmates are paid for by Boards of Guardians or parents. The remainder of the 223 persons, of whom 48 are over 16 years of age, are provided for by voluntary subscriptions. There can be no question at all that nine-tenths at least of these children would be chargeable to the public expense under one form or another if the institution ceased to exist. Would it not be reasonable to relieve voluntary associations which are carrying on work of this great public value from the strain of entirely maintaining those they benefit? The right policy, in the present tentative stage of provision for the feeble-minded, is for the State to encourage by every means in its power the voluntary efforts which have so far proved successful. There should be money enough and to spare forthcoming to equip a voluntary colony for the care of these poor creatures in every county in the kingdom. But the most enthusiastic hang back before the tremendous burden of supporting the inmates for life. Were it once understood that voluntary institutions equipped and in good working order would receive a grant in aid of each inmate, an enormous impulse would be given to this most necessary preventive work, and we venture to contend it would be carried through with greater economy and zeal than if it became a rate-supported task imposed on reluctant local authorities.

But the work to be of any real use must be permanent. At present parents have the power to remove their children at the age of sixteen from safe and happy surroundings and set them to impossible tasks in surroundings which speedily undo the toil of years. The time is not yet ripe, we honestly believe, for compulsory orders for the detention of all the feeble-minded. But might they not pass so far under the guardianship of the State that they should be left in colonies suited for them undisturbed by rapacious (often equally feeble-minded) parents? There will be no whole-hearted extension of this work until some security is given by the State that the success achieved in countless cases shall not be heartlessly destroyed. Four instances are quoted in the Sandlebridge report for last year in which children over sixteen have been removed at the mere claim of parents who may be, as they so often are, utterly unfit to take charge of creatures who will be all their lives only "children of a larger growth." We do not deny that the parent may have a sincere affection for the weak-minded child; but this affection is likely to tend to the assumption that the child is nearly normal, and to the encouragement of normal activities and of a free life which is dangerous alike to the child himself and to the community. However well meant on the part of both parents and authorities, it is cruel injustice, frequently to the feeble-minded being himself, and always to the nation. Is it not time that the State, having once recognised the weakness of any of its citizens, should claim the power of at least preventing such a weak-minded one having the opportunity of reproducing his kind?

### EUGENICS v. HYGIENICS.

On this unfortunately topical question it behoves medical men to speak cautiously, lest they be charged with preaching that which they do not practise. What, for instance, can be further from good eugenic practice than to mend a cleft palate? In the savage state a child thus affected would perish at the breast, but under civilisation it is patched up, and later is sent out into the world to favour, if Mr. Bland Sutton may be believed, the production of a hare-lipped and cleft-palated offspring; or worse still, if the heredity be indirect, an unfortunate being, who is the subject of hydrocephalus or congenital heart disease. This sickly parasite, costing much to rear, dies ere the threshold of working life is reached without having repaid one penny of its keep. Can a State thrive under such conditions?

The subject of development defects, indeed, is somewhat left out of count by ardent hygienists like Professor Moore, who in his last book * preaches the doctrine that we should do away with disease altogether. His plan is, first, to establish a national medical service, a public department staffed by whole time medical officers of health. By routine visitation of all houses in their districts they will instruct the masses in preventive hygiene and will nip chronic disease in the bud; by special visitation of suspected quarters during epidemics they will get their cases earlier and be enabled to take strict isolation precautions sooner than at present. The doctor will go to the hospital patient class instead of the hospital patient class coming, often too late, to the doctor. Secondly, he demands State medical insurance, and, thirdly, reform in the administration and practice of the Poor Law hospitals. The present practical monopoly of surgical handicraft is to be abolished by the short-period appointment of paid surgeons to the new hospitals. Thus the supply of surgeons will be increased, fees will sink proportionately, and the middle classes of society will benefit. Moreover, the lower ranks of the middle classes will be able to pay the reduced fees, and the question of hospital abuse will be settled—to the mutual advantage of the laity and of the medical profession. What a Utopia!

As has been noted, the present-day practitioner aims at patching up the individual, but this author at eliminating disease from the community. If, therefore, the eugenist looks askance at the former, how much more must he frown at Professor Moore! And in the latter case the divergence of opinion is indeed fundamental, for this author, like Dr. Archdall Reid and his forerunner, Henry George, seems to call in question the doctrine of survival of the fittest. Everything depends, he argues, on greater exposure to infection combined with greater lack of resistance, both proceeding from faulty hygienic environment. Disease and death fall alike on the intrinsically fit and the intrinsically unfit. In the present state of our knowledge this is an uncommonly bold thing to say.

The opposed school would argue that disease is necessary to eliminate the unfit, while a certain wastage of the fit accompanying such elimination, if brought to a minimum by hygienic legislation, must just be put up with. There is, however, an objection to this reasoning. Tuberculosis, for instance, in its commonest form does not affect the reproductive capacity of a man. The father may get consumption, but he does not die until children have been born to him. The children do not die until they in turn have propagated the species, and so the breed goes on. Thus tuberculosis, while it may kill degenerates in greater numbers than healthy individuals, has an indeterminate and only partial action. It kills so many healthy individuals along with the degenerates that its selective value is minimised. If the eugenist has little use for consumption he can have none at all for syphilis, and Professor Moore very rightly draws attention to the shrouded subject of the loss the State suffers from venereal disease. He pleads for preventive medicine; but, however easy and favourable conditions of life are made, there is no getting away from the fact that a race highly resistant to the health-lowering influences of urban life would be all-conquering. Still, the " pestilence walketh in darkness," and the race passing under its baneful influences may indeed tend to degeneracy rather than to strength. If the national health is to be conserved on lines which are the logical development of the modern trend of thought, then the schemes which the author brings into forcible prominence cannot be materially bettered.

A consideration of a less general nature falls to be mentioned in conclusion. As will have been gathered, a good many vested interests, of a good many kinds, are assailed pretty explicitly. Like all reformers, Professor Moore may be usefully reminded of Swift's dry warning, that "there is not in all nature another so callous and insensible a member as the world's posterior." However, if we read him aright, such warning will not surprise, certainly not frighten, him in the least.

* *The Dawn of the Health Age.* By B. Moore, D.Sc.

